# Serum Islet Cell Autoantibodies During Interferon α Treatment in Patients With HCV-Genotype 4 Chronic Hepatitis 

**DOI:** 10.1080/17402520600557867

**Published:** 2006-03

**Authors:** Gamal Badra, Imam Waked, Carlo Selmi, Saleh M. Saleh, Ahmed El-Shaarawy, Mahmoud Lotfy

**Affiliations:** 1 Department of Hepatology National Liver Institute Minufiya University Minufiya Egypt; 2 Division of Internal Medicine San Paolo School of Medicine University of Milan Milan Italy; 3 Division of Rheumatology, Allergy, and Clinical Immunology University of California Davis California USA; 4 Clinical Pathology Division National Liver Institute Minufiya University Minufiya Egypt; 5 Molecular and Cellular Biology Department Genetic Engineering and Biotechnology Research Institute Minufiya University Sadat City Minufiya Egypt

## Abstract

Chronic hepatitis C virus (HCV) infection is a leading cause of end-stage liver disease worldwide and HCV genotype 4 (HCV4) is predominant in African and Middle Eastern countries. It is well established that interferon-α (IFNa) treatment for HCV may trigger serum autoantibodies against pancreatic islet cells (ICA) in a subgroup of patients. Available data on the incidence of ICA during IFNa therapy for chronic HCV4 infection are not conclusive. We investigated the appearance of ICA in 40 naïve Egyptian patients (38 males, 32 ± 6 years) with histologically defined chronic HCV4 infection undergoing IFNa treatment at a dose of 9-million U/week for 24 weeks. Serum samples were collected at baseline and following IFNa therapy and ICA were detected using indirect immunofluorescence. Baseline evaluation indicated that 2/40 (5%) patients had detectable serum ICA. After the completion of the treatment scheme, 12/38 (32%) previously ICA negative patients became ICA positive; however, no patient developed impaired glucose tolerance (IGT) or diabetes during follow-up. In conclusion, we submit that IFNa treatment for chronic hepatitis C (CHC) may induce serum ICA in one-third of Egyptian patients with HCV4. These autoantibodies, however, do not lead to alterations in glucose metabolism.


*Clinical & Developmental Immunology*, March 2006; 13(1): 11–15

Taylor & Francis

Taylor & Francis Group

### Serum islet cell autoantibodies during interferon α treatment in patients with HCV-genotype 4 chronic hepatitis

GAMAL BADRA[1], IMAM WAKED[1], CARLO SELMI[2,3], SALEH M. SALEH[1], AHMED EL-SHAARAWY[4], & MAHMOUD LOTFY[5]

[1]*Department of Hepatology, National Liver Institute, Minufiya University, Minufiya, Egypt,* [2]*Division of Internal Medicine, San Paolo School of Medicine, University of Milan, Milan, Italy,* [3]*Division of Rheumatology, Allergy, and Clinical Immunology, University of California, Davis, California, USA,* [4]*Clinical Pathology Division, National Liver Institute, Minufiya University, Minufiya, Egypt, and* [5]*Molecular and Cellular Biology Department, Genetic Engineering and Biotechnology Research Institute, Minufiya University, Sadat City, Minufiya, Egypt*

**Abstract**
Chronic hepatitis C virus (HCV) infection is a leading cause of end-stage liver disease worldwide and HCV genotype 4 (HCV4) is predominant in African and Middle Eastern countries. It is well established that interferon-α (IFNa) treatment for HCV may trigger serum autoantibodies against pancreatic islet cells (ICA) in a subgroup of patients. Available data on the incidence of ICA during IFNa therapy for chronic HCV4 infection are not conclusive. We investigated the appearance of ICA in 40 naïve Egyptian patients (38 males, 32 ± 6 years) with histologically defined chronic HCV4 infection undergoing IFNa treatment at a dose of 9-million U/week for 24 weeks. Serum samples were collected at baseline and following IFNa therapy and ICA were detected using indirect immunofluorescence. Baseline evaluation indicated that 2/40 (5%) patients had detectable serum ICA. After the completion of the treatment scheme, 12/38 (32%) previously ICA negative patients became ICA positive; however, no patient developed impaired glucose tolerance (IGT) or diabetes during follow-up. In conclusion, we submit that IFNa treatment for chronic hepatitis C (CHC) may induce serum ICA in one-third of Egyptian patients with HCV4. These autoantibodies, however, do not lead to alterations in glucose metabolism.

**Keywords:** *Antiviral treatment, chronic hepatitis C, genotype 4, glucose tolerance*

### Introduction

Hepatitis C virus (HCV) infection is a leading cause of chronic hepatitis and it has been estimated to affect 170 million people worldwide (Lauer and Walker 2001). Prevalence rates for serum anti-HCV positivity in the general population range between 1% in North America (Alter 1997) to over 20% in endemic areas such as Egypt (Abdel-Aziz et al. 2000). Acute HCV infection leads to chronic hepatitis C (CHC) in the vast majority of cases thus increasing the risk of developing cirrhosis and eventually hepatocellular carcinoma (Alter and Seeff 2000, Liang et al. 2000). Six major HCV genotypes are known (Hoofnagle 2002) and significantly differ in their geographical distribution. HCV genotype 4 (HCV4) is predominant in Africa and the Middle East (McOmish et al. 1994) and is responsible for over 90% of infection cases in Egypt (Ray et al. 2000) while being rare in western countries.

Treatment regimens for CHC are based on interferon-α (IFNa) molecules usually in combination with ribavirin (Ahmed and Keeffe 1999). Standard dosing of non-pegylated IFNa molecules is 3 million U three times a week and were commonly used in Egypt until few years ago when validation of results with pegylated IFNa became available (Zeuzem et al. 2000). In all cases, however, IFNa is administered with ribavirin at daily doses of 1000/1200 mg. In 2–10% of cases, IFNa side effects may lead to dose modulation or cessation of therapy (Dusheiko 1997). Among serious side effects related to IFNa therapy, new-onset autoimmune manifestations are important

Correspondence: Dr Mahmoud, Molecular and Cellular Biology Department, Genetic Engineering and Biotechnology Research Institute, Minufiya University, PO 79, Sadat City, Minufiya, Egypt. E-mail: mlotfy2000@yahoo.com; mylotfy@mailer.menofia.edu.eg

ISSN 1740-2522 print/ISSN 1740-2530 online © 2006 Taylor & Francis
DOI: 10.1080/17402520600557867

causes of treatment discontinuation and often do not disappear after IFNa withdrawal. Such manifestations include the *de novo* appearance of serum autoantibodies against thyroid antigens (Kiehne et al. 1997) or pancreatic islet cells (ICA) (Wesche et al. 2001). The latter are particularly important since they significantly correlate with the onset of impaired glucose tolerance (IGT) or frank Diabetes mellitus (DM) (Batstra et al. 2001).

Data on ICA incidence in patients with HCV4 chronic infection undergoing IFNa treatment are not available. We herein report a serological study on 40 Egyptian patients with histologically defined CHC from HCV4 to define the risk in this subgroup of patients to develop serum ICA and/or IGT/DM during antiviral treatment.

### Materials and methods

### *Subjects*

We studied 40 patients (38 males, mean ± SD age 32 ± 6 years, range 24–52) with chronic HCV4 infection attending the outpatient clinic of the National Liver Institute at Minufiya University. Patients fulfilling the criteria for CHC diagnosis and treatment eligibility were consecutively enrolled between January 2002 and December 2003. The diagnosis of CHC was based on positive serum anti-HCV antibodies (Ortho Diagnostic Systems Inc., Raritan, NJ), positive serum HCV–RNA (Roche Molecular Systems, Inc., Pleasanton, CA) and by liver histology. All patients had HCV4. All patients had undergone a liver biopsy within 12 months prior to enrollment and histological features were consistent with the diagnosis of CHC (Gerber 1997). Fibrosis or cirrhosis was found in 20/40 (50%) cases. Patients with clinical signs and symptoms of decompensated cirrhosis (i.e. history of digestive bleeding from portal hypertension, ascites, jaundice or hepatic encephalopathy) were excluded from the study. No patient had been previously been treated with IFN for CHC.

Serum samples were obtained after overnight fasting before the beginning of treatment (baseline) as well as after 12 weeks of therapy and 24 weeks after treatment discontinuation for determination of ICA status. Sera were stored at −80° until use. Serum glucose levels after overnight fasting (FGL) and 2 h after a glucose load (PPGL) were determined at baseline and at week 24 using routine techniques. The study protocol respected the most recent Declaration of Helsinki (Edinburgh 2000), and all of the patients gave consent to the use of their sera and clinical data for research purposes after being informed about the nature of the study.

### *Antiviral treatment*

Forty patients were enrolled in the study and completed the six-month treatment. Twenty-four/40 (60%) and 16/40 (40%) patients were treated with recombinant IFNa–2b and IFNa–2a, respectively. In both groups, the dose was 3 million U subcutaneously three times a week and all patients also received a 1000/1200 mg daily dose of ribavirin. Treatment was discontinued after 24 weeks in all cases.

### *ICA detection*

Serum ICA were tested using indirect immunofluorescence on monkey pancreatic cells following the manufacturer's protocol (Biosystems, Barcelona, Spain). Sera showing light homogenous fluorescence in the cytoplasm of pancreatic islet cells were considered positive.

### *Statistical analysis*

Statistical analysis was performed using the Wilcoxon rank sum test for comparison of non-parametric variables and the McNemar test for the comparison of dichotomous variables. $P$ values $<0.05$ were considered as statistically significant. All comparisons were performed using SPSS v11 (SPSS Inc., Chicago, IL).

### Results

### *Response to treatment*

Table I summarizes the biochemical characteristics of patients at enrollment and following antiviral treatment.

Table I. Biochemical characteristics of patients with CHC before and after antiviral treatment.

| | Before treatment | After treatment | *P* value |
|---|---|---|---|
| Alanine aminotransferase (UI/l) | 90 ± 23 | 47 ± 29 | <0.01 |
| Aspartate aminotransferase (UI/l) | 66 ± 13 | 36 ± 22 | <0.01 |
| Total bilirubin (g/dl) | 1.28 ± 0.41 | 0.99 ± 0.25 | <0.05 |
| White blood cells (/μl) | 6626 ± 1086 | 5563 ± 0,865 | <0.01 |
| Platelets (/μl( $\times 10^3$) | 246 ± 51 | 196 ± 57 | <0.05 |
| Hemoglobin (g/dl) | 12.9 ± 0.6 | 11.1 ± 0.5 | <0.01 |
| HCV–RNA positive (n) | 40/40 (100%) | 10/40 (25%) | <0.01 |
| Fasting glucose level (mg/dl) | 87 ± 10 | 88 ± 7 | NS |
| Post prandial glucose level (mg/dl) | 100 ± 8 | 103 ± 11 | NS |

Continuous variables are expressed as mean ± standard deviation; NS, non significant

We observed statistically significant differences in white blood cell (WBC) and platelet counts, hemoglobin concentration, alanine (ALT) and aspartate (AST) aminotransferase, total bilirubin and albumin serum levels, and prothrombin time following IFNa + ribavirin therapy. Fasting and post-prandial blood glucose levels did not change significantly after treatment (87 ± 10 vs 88 ± 7 mg/dl and 100 ± 8 vs 103 ± 11 mg/dl, respectively; $P =$ non-significant for both comparisons). Serum HCV–RNA became undetectable at the end of treatment in 10/40 (25%) patients following 24 weeks of therapy.

### *Serum ICA*

Testing of ICA was carried out before treatment and 6 months after completion of therapy using indirect immunofluorescence; Figure 1 shows a representative positive serum pattern. Table II illustrates the changes of ICA positivity with treatment. Before antiviral therapy, only 2/40 (5%) patients were positive had detectable serum ICA and in both cases such reactivity was still found following antiviral treatment. Thirty-eight/40 (95%) patients were ICA-negative at baseline and 12/38 (32%) developed serum ICA at the end of treatment.

### *Glucose levels in serum ICA-based subgroups*

Table III illustrates fasting and post-prandial glucose levels in patients who developed serum ICA during antiviral treatment. We failed to observe significant changes in fasting (86 ± 13 mg/dl before treatment vs 86 ± 6 mg/dl after treatment; $P =$ NS) or post-prandial (100 ± 8 mg/dl before treatment vs 106 ± 14 mg/dl after treatment; $P =$ NS) glucose levels in the 12 patients who became serum ICA-positive after therapy with IFNa + ribavirin. Importantly, none of these patient developed IGT or DM. We also note that we failed to observe significant

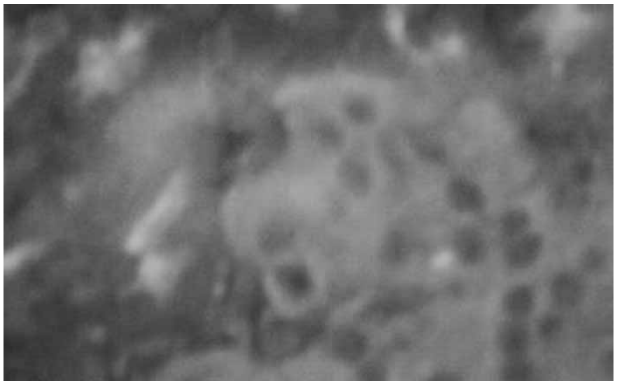

Figure 1. Detection of serum ICA using indirect immunofluorescence on monkey pancreatic cells. One representative serum showing a positive pattern indicated by light homogenous fluorescence in the cytoplasm of pancreatic islet cells is depicted.

Table II. Seroprevalence of ICA in patients with CHC before and after antiviral treatment.

| | *N* (%) |
|---|---|
| Negative before/negative after treatment | 26/40 (65%) |
| Positive before/positive after treatment | 2/40 (5%) |
| Negative before/positive after treatment | 12/40 (30%) |
| Positive before/negative after treatment | 0/40 |

clinical or biochemical differences when patients were subdivided based on the post-treatment serum ICA status (data not shown).

### Discussion

Antiviral treatments are being commonly used worldwide for CHC (EASL International Consensus Conference on Hepatitis C. Paris, 26–28, February 1999, Consensus Statement. European Association for the Study of the Liver 1999), thus making the definition of potential side effects a critical issue (Dusheiko 1997). The vast majority of available data, however, are based on patient series for Western Countries, thus possibly being poorly representative of other geographical areas where other HCV genotypes are predominant, as is the case for HCV4 in Egypt (Ray et al. 2000). We therefore investigated for the first time the effects of an IFNa-based antiviral treatment on serum ICA and glucose metabolism in patients with HCV4 chronic hepatitis. We report herein that despite a 30% incidence of serum ICA during a six-month treatment of IFNa and ribavirin, Egyptian patients with HCV4 chronic infection do not develop IGT or DM. We are aware that non-pegylated IFNa is currently no longer the first-line treatment of choice for CHC; however, we submit that it was still used at the time of patient enrollment in our center and believe that observations obtained with such treatment provide a good indication of potential side effects to be expected with the newer pegylated molecules.

Chronic HCV infection is a major cause of liver-related morbidity and mortality worldwide (Shepard et al. 2005). Current treatments based on IFNa induce a sustained virological response in 40–67% of patients (Teoh and Farrell 2004) but fail to clear the virus in a sustained fashion in a significant proportion of cases and are burdened by significant side effects. Moreover, the complexity related to the geographical distribution of HCV genotypes easily underscores the difficulties in global prevention and control of HCV by making an independent reproduction of data obtained in Western countries necessary for other geographical areas. The case of Egyptian patients with CHC, in this sense, is paradigmatic since HCV4 is strikingly predominant being responsible for over 90% of chronic infections (Ray et al. 2000). This specific

Table III. Fasting and post–prandial glucose levels (expressed as mean ± SD) before and after antiviral treatment in patients with CHC who developed ICA during IFNa therapy.

| Fasting glucose (mg/dl) | | Post-prandial glucose (mg/dl) | |
|---|---|---|---|
| Before treatment | After treatment | Before treatment | After treatment |
| 86 ± 13 | 86 ± 6 | 100 ± 8 | 106 ± 14 |

HCV genotype, in fact, has been found to present a peculiar pattern of progression and response to treatment (Nguyen and Keeffe 2005).

Autoimmune manifestations can be triggered by IFNa-based treatment for CHC in a significant proportion of cases (Fattovich et al. 1991, Fattovich et al. 1996). In particular, autoimmune thyroiditis is often diagnosed during therapy and is caused by the development of organ-specific autoantibodies (Marcellin et al. 1995). Other endocrine organs are also possible targets of IFNa-induced autoimmunity. The *de novo* appearance of serum antibodies to pancreatic antigens such as 21-hydroxylase (an autoantigen of the adrenal cortex), glutamate decarboxylase 65 (GAD65), or tyrosine phosphatase (IA2) has been reported in almost 10% of patients treated with IFNa (Wesche et al. 2001). The latter two autoantibodies are involved in the pathogenesis of type 1 diabetes. Further, several studies have demonstrated the development of serum autoantibodies and sporadic onset of type 1 diabetes in association with IFNa therapy (Fabris et al. 1998). Lastly, the appearance of two serum autoantibodies among ICA, GAD65 and IA2 in patients with CHC being treated with IFNa led to a considerable risk of progression to clinically overt diabetes (Wasmuth et al. 2001). An increased independent risk for DM development associated with chronic HCV infection was suggested based on clinical observations (Mason et al. 1999). Interestingly, however, no detectable GAD65 serum autoantibodies were found in untreated patients with CHC (Hieronimus et al. 1997), nor endocrine autoimmunity was induced by IFNa in patients with chronic hepatitis B infection (Fattovich et al. 1991). Taken altogether, these lines of evidence indicate that IFNa-based antiviral treatment and CHC might be necessary but not sufficient conditions for the induction of pancreatic autoimmunity.

As discussed above, HCV4 is the most common genotype involved in Egyptian cases of CHC. Such genotype has several peculiar characteristics compared to other genotypes. In fact, HCV4 infection appears to be most commonly secondary to iatrogenic transmission (Koshy et al. 2000, 2002) while also presenting a relatively low response rate to both non-pegylated (el-Zayadi et al. 1996) and pegylated (Legrand-Abravanel et al. 2005) IFNa. These observations constitute the bases for the recognized need of a separate analysis of HCV4 in clinical studies, commonly underrepresented in larger trials (Manns et al. 2001, Fried et al. 2002). Based on our data, however, we submit that the induction of pancreatic autoimmunity appears to follow a somehow peculiar pattern in HCV4-infected patients treated with IFNa compared to other genotypes. First, we report that the prevalence of serum ICA in untreated patients with CHC is higher than observed in other HCV genotypes, being 5% (2/40 in our series), but these cases do not present IGT or DM, similar to previous reports that included a small proportion of HCV4 (Betterle et al. 2000). Further, we observed that in both cases such reactivity was still present after IFNa treatment, thus somehow differing from the titer increase reported by others (Betterle et al. 2000). Second, we submit that the appearance of serum ICA following IFNa treatment should be expected in approximately one-third of patients with HCV4 chronic infection. The clinical impact of these autoantibodies, however, appear to be minor since no case of IGT or DM was found, similar to observations in other genotypes (Betterle et al. 2000).

In conclusion, we submit that pancreatic-specific autoimmunity is not a rare occurrence both before and after IFNa treatment in Egyptian patients with HCV4 chronic infection. This assumption warrants a further confirmation based on larger studies on well-defined middle-eastern patients and the use of pegylated IFNa. Until these data become available, however, we suggest that the screening of patients with CHC before and after antiviral therapy might prove beneficial for the early diagnosis of pancreatic autoimmune manifestations.

### References

Abdel-Aziz F, Habib M, Mohamed MK, Abdel-Hamid M, Gamil F, Madkour S, Mikhail NN, Thomas D, Fix AD, Strickland GT, Anwar W, Sallam I. 2000. Hepatitis C virus (HCV) infection in a community in the Nile Delta: Population description and HCV prevalence. Hepatology 32:111–115.

Ahmed A, Keeffe EB. 1999. Overview of interferon therapy for chronic hepatitis C. Clin Liver Dis 3:757–773.

Alter HJ, Seeff LB. 2000. Recovery, persistence, and sequelae in hepatitis C virus infection: A perspective on long-term outcome. Semin Liver Dis 20:17–35.

Alter MJ. 1997. The epidemiology of acute and chronic hepatitis C. Clin Liver Dis 1:559–568, vi–vii.

Batstra MR, Aanstoot HJ, Herbrink P. 2001. Prediction and diagnosis of type 1 diabetes using beta-cell autoantibodies. Clin Lab 47:497–507.

Betterle C, Fabris P, Zanchetta R, Pedini B, Tositti G, Bosi E, de Lalla F. 2000. Autoimmunity against pancreatic islets and other tissues before and after interferon-α therapy in patients with hepatitis C virus chronic infection. Diabetes Care 23: 1177–1181.

Dusheiko G. 1997. Side effects of α interferon in chronic hepatitis C. Hepatology 26:112S–121S.

1999. EASL International Consensus Conference on Hepatitis C. Paris, 26-28, February 1999, Consensus Statement. European Association for the Study of the Liver. J Hepatol 30:956–961.

el-Zayadi A, Simmonds P, Dabbous H, Prescott L, Selim O, Ahdy A. 1996. Response to interferon-α of Egyptian patients infected with hepatitis C virus genotype 4. J Viral Hepatol 3:261–264.

Fabris P, Betterle C, Greggio NA, Zanchetta R, Bosi E, Biasin MR, de Lalla F. 1998. Insulin-dependent diabetes mellitus during α-interferon therapy for chronic viral hepatitis. J Hepatol 28:514–517.

Fattovich G, Betterle C, Brollo L, Pedini B, Giustina G, Realdi G, Alberti A, Ruol A. 1991. Autoantibodies during α-interferon therapy for chronic hepatitis B. J Med Virol 34:132–135.

Fattovich G, Giustina G, Favarato S, Ruol A. 1996. A survey of adverse events in 11,241 patients with chronic viral hepatitis treated with α-interferon. J Hepatol 24:38–47.

Fried MW, Shiffman ML, Reddy KR, Smith C, Marinos G, Goncales FL, Jr, Haussinger D, Diago M, Carosi G, Dhumeaux D, Craxi A, Lin A, Hoffman J, Yu J. 2002. Peginterferon alfa-2a plus ribavirin for chronic hepatitis C virus infection. N Engl J Med 347:975–982.

Gerber MA. 1997. Histopathology of HCV infection. Clin Liver Dis 1:529–541, vi.

Hieronimus S, Fredenrich A, Tran A, Benzaken S, Fenichel P. 1997. Antibodies to GAD in chronic hepatitis C patients. Diabetes Care 20:1044.

Hoofnagle JH. 2002. Course and outcome of hepatitis C. Hepatology 36:S21–S29.

Kiehne K, Kloehn S, Hinrichsen H, Gallwitz B, Monig H. 1997. Thyroid autoantibodies and thyroid dysfunction during treatment with interferon-α for chronic hepatitis C. Endocrine 6:231–234.

Koshy A, Madda JP, Marcellin P, Martinot M. 2002. Treatment of hepatitis C virus genotype 4-related cirrhosis: Ribavirin and interferon combination compared with interferon alone. J Clin Gastroenterol 35:82–85.

Koshy A, Marcellin P, Martinot M, Madda JP. 2000. Improved response to ribavirin interferon combination compared with interferon alone in patients with type 4 chronic hepatitis C without cirrhosis. Liver 20:335–339.

Lauer GM, Walker BD. 2001. Hepatitis C virus infection. N Engl J Med 345:41–52.

Legrand-Abravanel F, Nicot F, Boulestin A, Sandres-Saune K, Vinel JP, Alric L, Izopet J. 2005. Pegylated interferon and ribavirin therapy for chronic hepatitis C virus genotype 4 infection. J Med Virol 77:66–69.

Liang TJ, Rehermann B, Seeff LB, Hoofnagle JH. 2000. Pathogenesis, natural history, treatment, and prevention of hepatitis C. Ann Int Med 132:296–305.

Manns MP, McHutchison JG, Gordon SC, Rustgi VK, Shiffman M, Reindollar R, Goodman ZD, Koury K, Ling M, Albrecht JK. 2001. Peginterferon alfa-2b plus ribavirin compared with interferon α-2b plus ribavirin for initial treatment of chronic hepatitis C: A randomised trial. Lancet 358:958–965.

Marcellin P, Pouteau M, Benhamou JP. 1995. Hepatitis C virus infection, α-interferon therapy and thyroid dysfunction. J Hepatol 22:364–369.

Mason AL, Lau JY, Hoang N, Qian K, Alexander GJ, Xu L, Guo L, Jacob S, Regenstein FG, Zimmerman R, Everhart JE, Wasserfall C, Maclaren NK, Perrillo RP. 1999. Association of diabetes mellitus and chronic hepatitis C virus infection. Hepatology 29:328–333.

McOmish F, Yap PL, Dow BC, Follett EA, Seed C, Keller AJ, Cobain TJ, Krusius T, Kolho E, Naukkarinen R, et al. 1994. Geographical distribution of hepatitis C virus genotypes in blood donors: An international collaborative survey. J Clin Microbiol 32:884–892.

Nguyen MH, Keeffe EB. 2005. Chronic hepatitis C: Genotypes 4 to 9. Clin Liver Dis 9:411–426, vi.

Ray SC, Arthur RR, Carella A, Bukh J, Thomas DL. 2000. Genetic epidemiology of hepatitis C virus throughout Egypt. J Infect Dis 182:698–707.

Shepard CW, Finelli L, Alter MJ. 2005. Global epidemiology of hepatitis C virus infection. Lancet Infect Dis 5:558–567.

Teoh NC, Farrell GC. 2004. Management of chronic hepatitis C virus infection: A new era of disease control. Int Med J 34:324–337.

Wasmuth HE, Stolte C, Geier A, Gartung C, Matern S. 2001. Induction of multiple autoantibodies to islet cell antigens during treatment with interferon α for chronic hepatitis C. Gut 49:596–597.

Wesche B, Jaeckel E, Trautwein C, Wedemeyer H, Falorni A, Frank H, von zur Muhlen A, Manns MP, Brabant G. 2001. Induction of autoantibodies to the adrenal cortex and pancreatic islet cells by interferon α therapy for chronic hepatitis C. Gut 48:378–383.

Zeuzem S, Feinman SV, Rasenack J, Heathcote EJ, Lai MY, Gane E, O'Grady J, Reichen J, Diago M, Lin A, Hoffman J, Brunda MJ. 2000. Peginterferon alfa-2a in patients with chronic hepatitis C. N Engl J Med 343:1666–1672.

